# Endourological Management of Urolithiasis in Donor Kidneys prior to Renal Transplant

**DOI:** 10.5402/2011/242690

**Published:** 2011-06-22

**Authors:** Nikhil Vasdev, John Moir, Muhammed T. Dosani, Robert Williams, Naeem Soomro, David Talbot, David Rix

**Affiliations:** ^1^Department of Urology, Freeman Hospital, Newcastle upon Tyne NE7 7DN, UK; ^2^Department of Renal Transplant, Freeman Hospital, Newcastle upon Tyne NE7 7DN, UK; ^3^Department of Radiology, Freeman Hospital, Newcastle upon Tyne NE7 7DN, UK

## Abstract

*Background*. We present our centres successful endourological methodology of ex vivo ureteroscopy (EVFUS) in the management of these kidneys prior to renal transplantation. *Patient and Methods*. A retrospective analysis was performed of all living donors (*n* = 157) identified to have asymptomatic incidental renal calculi from January 2004 until December 2008. The incidence of asymptomatic renal calculi was 3.2% (*n* = 5). Donors were subdivided into 2 groups depending on whether theydonated the kidney with the renal calculus (Group 1) versus the opposite calculus-free kidney (Group 2). *Results*. All donors in Group 1 underwent a left laparoscopic donor nephrectomy. The calculi were extracted in all 3 cases using a 7.5 Fr flexible ureteroscope either prior to transplant (*n* = 2) or on revascularization (*n* = 1). There were no urological complications in either group. At a mean followup at 64 months there was no recurrent calculi formation in the recipient in Group 1. However, 1 recipient formed a calculus in group 2 at a follow up of 72 months. *Conclusions*. Renal calculi can be successfully retrieved during living-related transplantation at the time of transplant itself using EVUS. This is technically feasible and is associated with no compromise in ureteral integrity or renal allograft function.

## 1. Introduction

Within the United Kingdom (UK), living-related donor nephrectomies (LRDNs) are becoming common. The most important constituent for a successful LRDN and subsequent transplants is the synchronous management of the donor and recipients by a multidisciplinary team approach involving urologist, renal transplant surgeons, nephrologist, and radiologist.

The long-term graft functions following living-related renal transplant (LRRT) are superior in comparison to cadaveric transplantation at all ages, particularly in the very young recipients [1, 2]. The incidence of LRRT varies from country to country. In the UK the incidence was 16% [3], whereas in North America 49% of paediatric transplants tend to be from living donors [2]. In Scandinavia the incidence of LRRT is as high as 86% [4]. Within the UK a total of 1,140 people were approved to become living donors during 2009-2010; this was 92 more than the year before and up from 342 in 2006-2007 which is a rise of 333% in four years [5].

Urolithiasis in donor kidneys is a relatively uncommon clinical entity and has an annual incidence of less than 1% [6, 7]. Two decades ago, urolithiasis within the donor kidney was deemed an absolute contraindication for donation as this was theoretically associated risk of postoperative allograft dysfunction due to urolithiasis when transplanted into the recipient [8]. From the donor's perspective, there is also an additional risk of future stone formation in the remaining kidney which could lead to possible sequelae of urolithiasis such as obstruction uropathy, urinary tract infections (UTI), sepsis, and end-stage renal disease (ESRD) [9].

With the current advances in endourology and radiological imaging, donor kidney urolithiasis can be accurately assessed on preoperative computerized tomography (CT) scans. The anatomy of the remaining kidney can also be assessed for similar abnormalities. Current recommendations for incidentally diagnosed renal calculi in donors include a thorough metabolic screen to ensure that the kidney is deemed suitable for transplantation if it contains a small asymptomatic stone [10, 11]. The current strategies in the literature for the management of urolithiasis in donor kidneys are preoperative treatment with extracorporeal shock wave lithotripsy (ESWL) or percutaneous nephrolithotomy (PCN) [12–16]. 

The decision as to whether the donor should be left with the stone-bearing kidney or whether this kidney should be used as a graft for the transplant is based on a detailed discussion process involving the donor and the multidisciplinary team of clinicians involved in the donor's care which include the urologist, renal transplant surgeons, nephrologist, and radiologist. 

**Figure 1:**
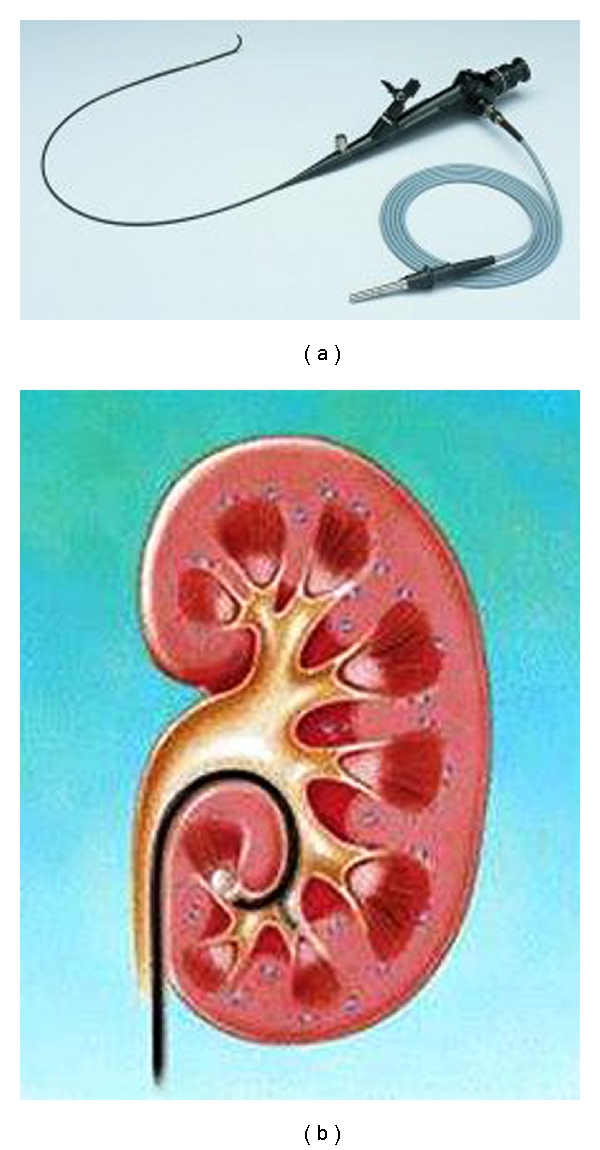
7.5 Fr flexible ureteroscope (a) being used for stone extraction (b).

We describe our successful endourological strategy of using an ex vivo 7.5 French (Fr) flexible ureteroscope (EVFUS) (Figure 1) and an Escape Nitinol Stone Retrieval Device to successfully extract renal calculi at the time of renal transplantation. We also review current additional strategies in the management of these rare and challenging cases in the current literature. 

## 2. Patients and Methods

A retrospective analysis was performed of all living donors identified to have asymptomatic incidental renal calculi on preoperative CT from January 2004 until December 2008. At our centre we performed a total of 512 renal transplants during this period amongst which 30.7% (*n* = 157) were LRRT. Amongst this cohort the incidence of asymptomatic renal calculi was 3.2% (*n* = 5). 

The exact location, size, and anatomy of the kidney bearing the stone were delineated prior to the planned LRDN. In 2 of the donors the opposite kidney was removed, and in the remaining 3 donors stone extraction was performed using a 7.5 Fr flexible ureteroscope at the time of transplantation using the following technique.

**Figure 2:**
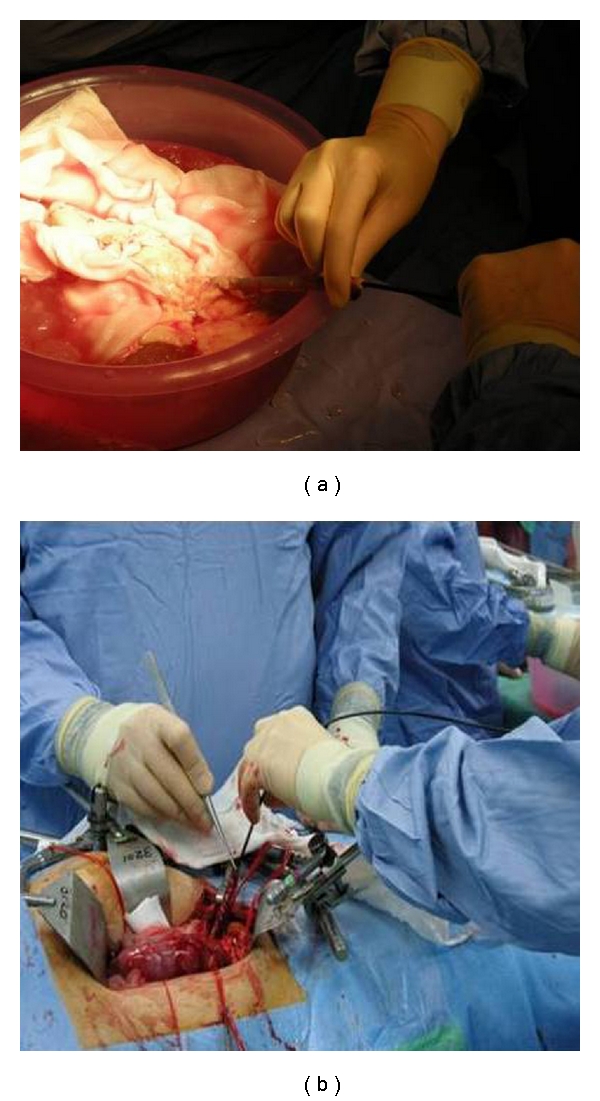
Use of the flexible ureteroscope in the Bench technique (a) and On-Table technique (b).

In our technique described, a 7.5 Fr flexible ureteroscope is placed into the graft ureter under low pressure irrigation to achieve the renal calculus which is then extracted using the Escape Nitinol Stone Retrieval Device. This technique is the Bench technique and has the potential disadvantage of prolonging warm ischemia time (WIT) (Figure 2(a)). The modification of the technique involves the use of the 7.5 Fr flexible ureteroscope when the renal donor graft has been revascularized into the recipient (On-Table technique) (Figure 2(b)).

## 3. Results

Amongst the 5 donors identified with asymptomatic urolithiasis, we used 3 of these kidneys for renal transplantation. In the remaining 2 patients the kidneys with urolithiasis were left in situ. The donors themselves in both these cases decided against donating their kidney with urolithiasis. In this section we divided these donors into those who donated their kidney with urolithiasis (Group 1, *n* = 3) and those who voluntarily decided to donate their opposite kidney with no urolithiasis (Group 2, *n* = 2). 

The mean age of donors in Group 1 was 47.6 years (range 46–50) and in Group 2 was 43.5 years (range 40–47) (*P* = NS). The age of the recipients in Group 1 was 29.3 years (range 22–51) and in Group 2 was 44 years (range 28–60) (*P* = NS). There was no difference in preoperative American Society of Anaesthesiologists (ASA) score and Glomerular filtration rate (GFR) or significant difference in split renal function on [99mTc]-DTPA renography in donors within both groups. Donors in both groups had detailed preoperative investigations for urolithiasis which included serum biochemical analysis (ionized calcium, uric acid, parathyroid hormone levels, and creatinine) and urinary electrolyte analysis. All investigations in both groups were normal. 

**Figure 3:**
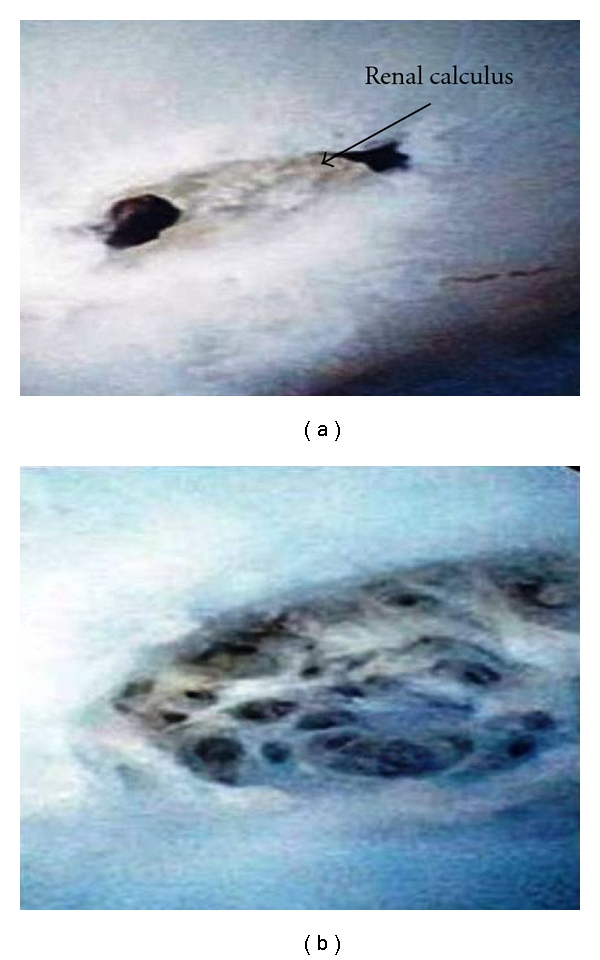
Flexible ureteroscopic view of calculi within the renal donor graft before (a) and after extraction (b).

Within Group 1, 3 donors were diagnosed with asymptomatic urolithiasis present within their left kidney. The calculi were lower pole (*n* = 2) and mid pole (*n* = 1) in the left kidney. The mean size of the renal calculi identified was 3.6 mm (range 3–5). The mean split function on the preoperative renogram was 49% (range of the left kidney 46–53). Following donor nephrectomy, these calculi were extracted using our technique described using the 7.5 Fr flexible ureteroscope and the “Bench technique” in 1 renal graft prior to transplant with a WIT of 5 minutes. In the remaining 2 cases the calculi were extracted using the “On-Table” technique. Biochemical analysis of the extracted calculi confirmed calcium oxalate with a mean composition of 80% and calcium phosphate of 20%. The view of the major calyx before and after stone extraction is demonstrated in Figure 3. At a mean followup of 64 months (range 48–84) both the donor and recipients are stone free. Additionally, there were no complications in the graft function and more importantly no urological complications such as ischemic strictures, anastomotic stenosis, or urinary leak.

In Group 2, both patients were diagnosed with right-sided asymptomatic urolithiasis and voluntary donated their left kidney after detailed counselling. The initial recipient in this group developed a renal calculus measuring 7 × 2 mm requiring a ureteroscopic stone extraction at 48-month followup. Biochemical analysis of the extracted calculus confirmed a composition of calcium phosphate 77% and calcium oxalate 23%. At a further followup of 60 months from initial transplant and 12 months following ureteroscopic stone extract, the recipient is stone-free. The remaining recipient in this group is stone-free at a followup of 72 months following transplant.

## 4. Discussion

Urolithiasis is common and can be asymptomatic in donors. Our ex vivo ureteroscopic management of asymptomatic calculi using the flexible ureteroscope and stone retrieval using the Escape Nitinol Stone Retrieval Device is safe, successful, and associated with no long-term graft-related complications or urological complications which include ischemic/anastomotic strictures or urinary leak on transplantation. 

In LRRT the donor's long-term health is of utmost priority. The current literature suggests that the incidence of stone progression in patients with asymptomatic calculi can be as high as 77% with the need for urological intervention being 26% [17]. Hence, it is important to evaluate donors with asymptomatic renal calculi for metabolic abnormalities in order to correct these and prevent the further risk of renal calculi formation. Those donors who are not at a proven risk for recurrent stone formation may be suitable for donating their kidney with renal calculus if the current stone is less than 15 mm and the kidney is anatomically suitable for transplantation [17–19].

In our series, one recipient developed a further calculus despite having a non-stone-bearing kidney transplanted. The incidence of renal calculus formation following renal transplantation in recipients is approximately 5-6% [20]. In this series a cohort of 710 renal transplant recipients were evaluated for the risk transplant graft renal calculus formation over a duration of 4 years. The incidence of renal calculus formation within this cohort was 6% (44). Interestingly, a renal calculus disease in the donors was a contraindication to using the kidney during renal transplantation within this series [20].

Van Gansbeke et al. reported on one donor who developed allograft failure secondary to renal calculus formation [8]. Qazi et al. discuss two cases which also developed renal calculi within their renal graft following transplantation [18]. All three cases [8, 18] required emergency surgical intervention, and none of the cases were associated with long-term urological complication or graft failure. There is particular concern regarding renal graft calculus formation as these can be difficult to diagnose as patients do not present with typical symptoms of “loin to groin” pain when they develop. The manifestations can present a few days or weeks following the onset of calculi formation which includes severe sepsis secondary to obstructive uropathy or loss of graft function in severe cases, ureteral stricture, or urinary tract infection [12, 14, 15, 19]. Postoperative allograft dysfunction as result of an obstructive uropathy can be delayed with potentially disastrous, and yet avoidable, consequences. 

In previous studies stones have been managed in the postoperative period using ESWL or PCN prior to transplant [12–16]. These techniques are advocated to be a safe and satisfactory approach in these papers. However, both techniques are associated with the risk of stone-associated complications in the early postoperative period, which could be potentially avoided if the stones had been removed prior to implantation. 

There are also possible difficulties associated with ESWL on a transplanted kidney, as found by Bhadauria et al. [19]. The authors commented on difficulty in localising calculi or small residual fragments accurately prior to transplant. In this series a high residual stone rate (25 to 40%) was noted for lower caliceal stones, whilst periureteral fibrosis was thought to be associated with making the ureter less pliable and thus manipulation with subsequent possible EVFUS difficult [19].

Rashid et al. described 10 cases of EVFUS for the treatment of donor calculi [9]. The authors found the technique easier as the removed kidney no longer exhibited the normal anatomical narrowing of the ureter at the iliac vessels and the ureterovesical junction. Furthermore the kidney could be manipulated to allow easier access to all the calices. In their study all but one stone was successfully treated and/or removed, with diameters ranging from 1 to 8 mm. No intraoperative or postoperative complications were experienced. There was no stone recurrence in donors (average followup of 36.4 months) or recipients (33.2), as well as no delayed ureteral stricture formation.

Trivedi et al. had an equally successful experience with EVFUS in 3 cases [21]. Although one patient required posttransplantation ESWL for migration of a small lower caliceal calculus into the upper ureter found on postoperative imaging, there were no significant ureteral complications and at mean followup of 2.2 years no new stone had formed in either the recipient or donor. 

For donors who have suffered from calculi due to pelviureteric junction obstruction, resulting in persisting hydronephrosis, there is the additional management option of a pyeloplasty at the time of transplant. We have performed one such procedure at our centre [22]. The donor had a mildly hydronephrotic right kidney with a GFR of 107 mL per minute but only 32% function from the right kidney (right kidney GFR: 35.3). The procedure was performed without complication by anastomosing the pelvis of the transplant to the recipient ureter, end to end over a ureteric stent, which was removed 4 weeks later.

Based on our experience, we recommend that EVFUS is a feasible and safe technique for stone extraction in donor kidneys prior to LRRT. The procedure can be performed by either the “Bench technique” or “On-Table technique,” the main difference being the initial technique being performed prior to renal allograft revascularisation versus the latter which is performed following revascularisation.

## 5. Conclusion

In conclusion the donor is the most important in the donor/recipient combination and the choice of kidney should reflect this. Therefore the kidney with the stone should be donated leaving the donor with the healthiest kidney. In order to do this strategies should be evolved to manage the stone in the transplanted kidney. One such strategy is to utilise ureteroscopy and stone removal during the ex vivo stage.

##  Conflict of Interests

The authors declare no conflict of interests.

## Abbreviations

ASA: American Society of Anaesthesiologists
CT: Computerized tomography
ESWL: Extracorporeal shock wave lithotripsy
EVFUS: Ex vivo ureteroscopy
GFR: Glomerular filtration rate
LRDN: Living-related donor nephrectomy
LRRT: Living-related renal transplant
PCN: Percutaneous nephrolithotomy
WIT: Warm ischemia time
UK: United Kingdom.
